# Clinical application of intrathecal gadobutrol for assessment of cerebrospinal fluid tracer clearance to blood

**DOI:** 10.1172/jci.insight.147063

**Published:** 2021-05-10

**Authors:** Per K. Eide, Espen Mariussen, Hilde Uggerud, Are H. Pripp, Aslan Lashkarivand, Bjørnar Hassel, Hege Christensen, Markus Herberg Hovd, Geir Ringstad

**Affiliations:** 1 Department of Neurosurgery, Oslo University Hospital, Rikshospitalet, Oslo, Norway.; 2 Institute of Clinical Medicine, Faculty of Medicine, University of Oslo, Oslo, Norway.; 3 Norwegian Institute for Air Research, Kjeller, Norway.; 4 Oslo Centre of Biostatistics and Epidemiology, Research Support Services,; 5 Department of Neurohabilitation, and; 6 Section for Pharmacology and Pharmaceutical Biosciences, Department of Pharmacy, University of Oslo, Oslo, Norway.; 7 Division of Radiology and Nuclear Medicine, Department of Radiology, Oslo University Hospital, Rikshospitalet, Oslo, Norway.

**Keywords:** Neuroscience, Alzheimer disease, Diagnostics, Neurodegeneration

## Abstract

**BACKGROUND:**

Methodology for estimation of cerebrospinal fluid (CSF) tracer clearance could have wide clinical application in predicting excretion of intrathecal drugs and metabolic solutes from brain metabolism and for diagnostic workup of CSF disturbances.

**METHODS:**

The MRI contrast agent gadobutrol (Gadovist) was used as a CSF tracer and injected into the lumbar CSF. Gadobutrol is contained outside blood vessels of the CNS and is eliminated along extravascular pathways, analogous to many CNS metabolites and intrathecal drugs. Tracer enrichment was verified and assessed in CSF by MRI at the level of the cisterna magna in parallel with obtaining blood samples through 48 hours.

**RESULTS:**

In a reference patient cohort (*n* = 29), both enrichment within CSF and blood coincided in time. Blood concentration profiles of gadobutrol through 48 hours varied between patients diagnosed with CSF leakage (*n* = 4), idiopathic normal pressure hydrocephalus dementia (*n* = 7), pineal cysts (*n* = 8), and idiopathic intracranial hypertension (*n* = 4).

**CONCLUSION:**

Assessment of CSF tracer clearance is clinically feasible and may provide a way to predict extravascular clearance of intrathecal drugs and endogenous metabolites from the CNS. The peak concentration in blood (at about 10 hours) was preceded by far peak tracer enhancement at MRI in extracranial lymphatic structures (at about 24 hours), as shown in previous studies, indicating a major role of the spinal canal in CSF clearance capacity.

**FUNDING:**

The work was supported by the Department of Neurosurgery, Oslo University Hospital; the Norwegian Institute for Air Research; and the University of Oslo.

## Graphical abstract

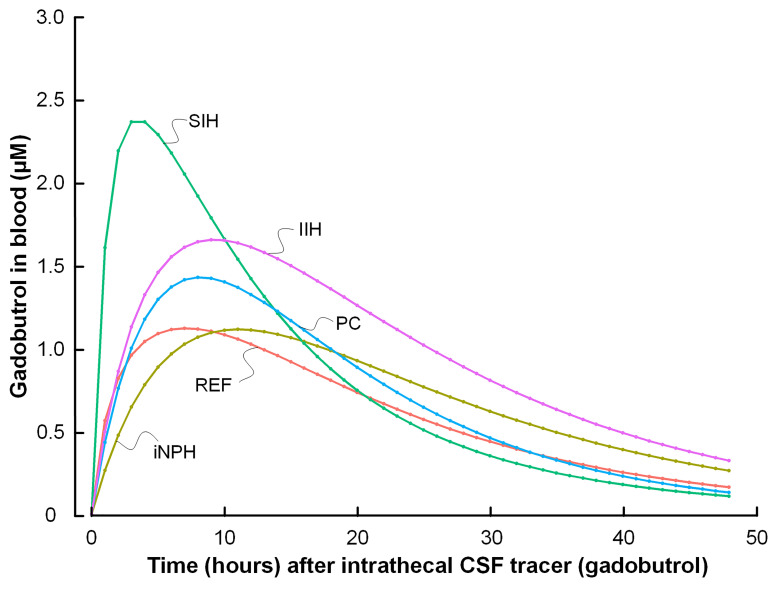

## Introduction

Methodology for estimation of cerebrospinal fluid (CSF) molecular clearance would be beneficial in several ways. Intrathecal administration of drugs is presently emerging as a promising route for treatment of a range of CNS diseases, including neuroinflammatory, neurodegenerative, neurooncologic, and neurovascular diseases (1–5). Although effective drug delivery to the CNS is restricted by the blood-brain barrier (BBB) during systemic administration (6), the human extravascular compartment of all brain regions is continuous with CSF and may be reached with intrathecal administration, bypassing the BBB. Remarkably, no test to predict clearance of substances from CSF has been established in the clinic.

The ability to predict clearance of endogenous molecules through CSF would also be crucial in assessment of neurodegenerative disease and dementia, and particularly in the preclinical phase of disease. Recent studies on animals and humans point at an instrumental role in extravascular clearance of metabolic waste products from CNS and its dependency on age and sleep (7, 8) for disease development. Examples of such metabolic by-products are amyloid-β (Aβ) and tau, which are found deposited in brain of subjects with neurodegenerative diseases, the most prevalent being Alzheimer’s disease. Both tau and some neurotoxic Aβ isoforms, e.g., pyroglutamate Aβ, pE3-Aβ, lack BBB transporters, and are cleared outside the BBB via the CSF and further to lymphatic routes (9). Attempts to assess impaired clearance capacity of Aβ and tau from brain, which would be particularly useful in the presymptomatic phase of the disease, have been limited mainly to measurements of levels in blood (10).

Here, we studied the clinical feasibility and utility of estimating CSF tracer clearance from the craniospinal compartment using the gadolinium-based (Gd-based) MRI contrast agent gadobutrol as CSF tracer. Gadobutrol is a hydrophilic, stable compound hypothesized to be excreted from CSF along clearance pathways similar to those of other drugs and endogenous metabolites not being cleared at the BBB. After intrathecal administration of the tracer, we consecutively measured blood concentration of Gd over time in parallel with standardized T1 MRI sequences for assessment of tracer enhancement in CSF. The CSF tracer clearance to blood was assessed in a reference patient cohort (REF) with no identified CSF disturbance, and in individuals with CSF disturbances, including a cohort with the dementia subtype idiopathic normal pressure hydrocephalus (iNPH).

## Results

### Patients.

This study included 52 individuals (Table 1). In 29 patients, denoted as REF subjects, no apparent CSF disturbance was identified and no intervention was indicated (Table 1). However, they are not healthy individuals. We also included subjects with various CSF disturbances, i.e., spontaneous intracranial hypotension (SIH), iNPH, pineal cysts (PCs), and idiopathic intracranial hypertension (IIH; Table 1).

### Tracer enrichment in CSF and elimination to blood in references.

**Figure 1:**
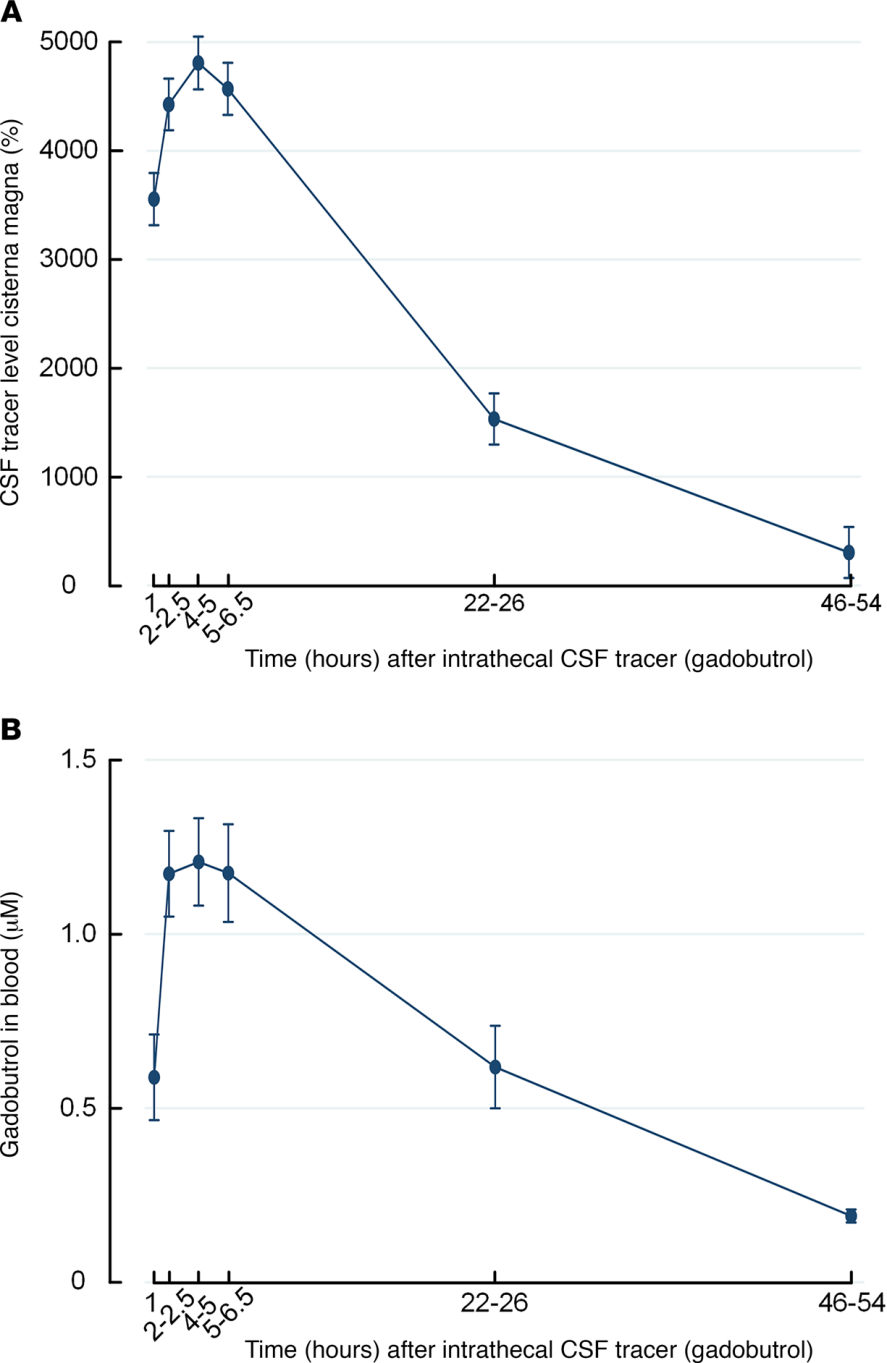
Trend plots of cerebrospinal fluid tracer levels in cisterna magna and blood of reference cohort subjects. (**A**) Enrichment of tracer in cerebrospinal fluid (CSF) of cisterna magna is shown as trend plot of percentage change in MRI signal unit ratio. (**B**) Clearance of tracer to blood is presented as concentrations of gadobutrol in whole blood. Trend plots are shown as mean ± SEM.

Following lumbar injection of CSF tracer (0.5 mmol gadobutrol), the tracer enriched CSF within the cisterna magna (Supplemental Figure 1; supplemental material available online with this article; https://doi.org/10.1172/jci.insight.147063DS1). For the REF group, tracer enrichment in CSF as shown by MRI (Figure 1A) and levels of gadobutrol in blood (Figure 1B) trended similarly over time.

### Association between MRI-based assessment of CSF tracer enrichment and blood concentrations of CSF tracer.

**Figure 2:**
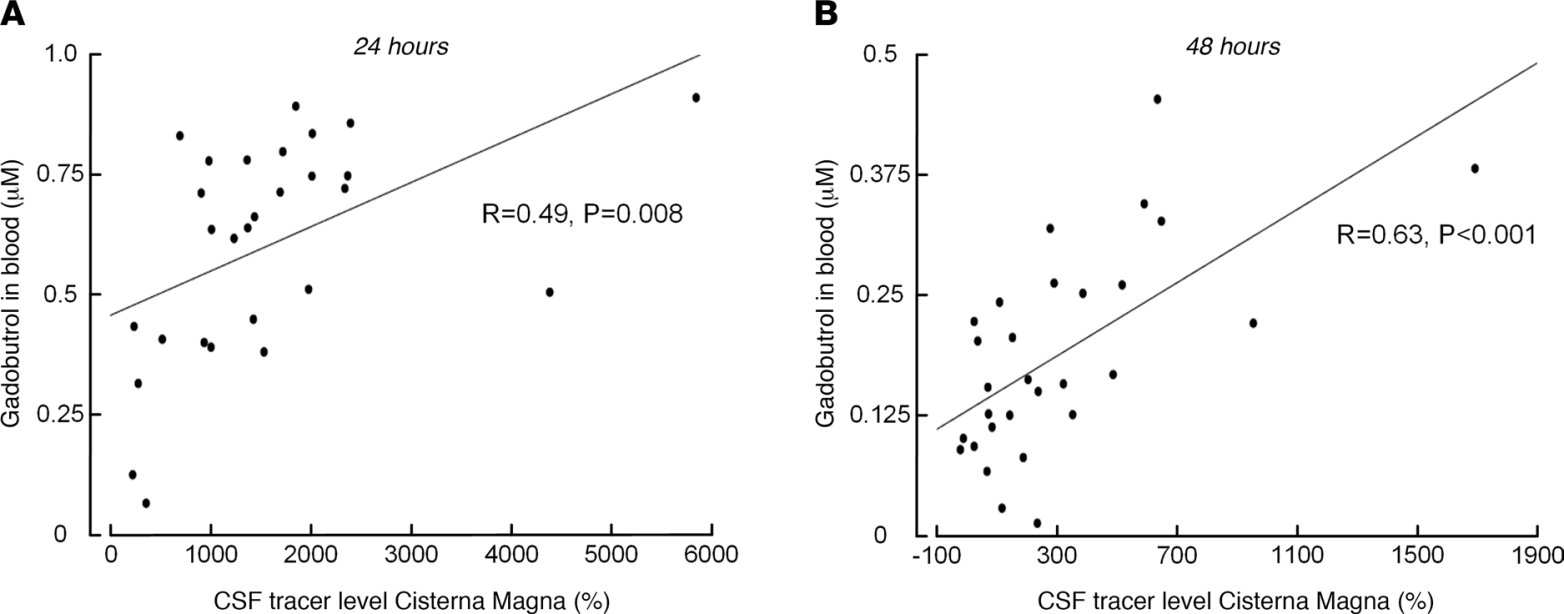
Correlation between enrichment of CSF tracer in cisterna magna assessed by MRI and tracer concentrations in blood at different time points in reference cohort subjects. (**A**) Twenty-four hours after intrathecal tracer administration, there was a significant positive correlation between tracer enrichment within CSF spaces of cisterna magna and blood concentration of the tracer. (**B**) Forty-eight hours after intrathecal tracer administration, there was also a significant positive correlation between tracer enrichment within CSF spaces of cisterna magna and blood concentration of the tracer. Each scatter plot presents fit lines and Pearson’s correlation coefficients with significance levels. We verified that the correlation between the variables showed a positive linear relationship.

There was a significant positive linear correlation between tracer enrichment in CSF and tracer concentrations in blood in REF subjects after 24 hours (Figure 2A) and after 48 hours (Figure 2B). The same results were obtained when pooling all 52 participants in the correlation analysis. Thus, at late time points, increased tracer levels in cisterna magna were associated with higher blood concentrations.

### Association between age and MRI-based CSF tracer enrichment and blood concentrations of CSF tracer.

Among REF subjects, there was a significant positive correlation between age and MRI CSF tracer enrichment at 48 hours, and between age and blood concentrations at 24 and 48 hours (Table 2). We found comparable results when including the entire cohort of 52 individuals in the analysis. Accordingly, the association between tracer enrichment in CSF and concentration in blood was strongest at late time points and increasingly with age.

In REF subjects, age was negatively correlated with glomerular filtration rate (GFR) (Pearson’s correlation coefficient –0.71, *P* < 0.001), but GFR was neither correlated with tracer enrichment in cisterna magna nor correlated with tracer concentrations in blood. The GFR is the most important measure for clearance function of the kidneys (11) and is used on a routine clinical basis.

### Comparisons of tracer enrichment in CSF and blood concentrations between references and patients with CSF disturbances.

**Figure 3:**
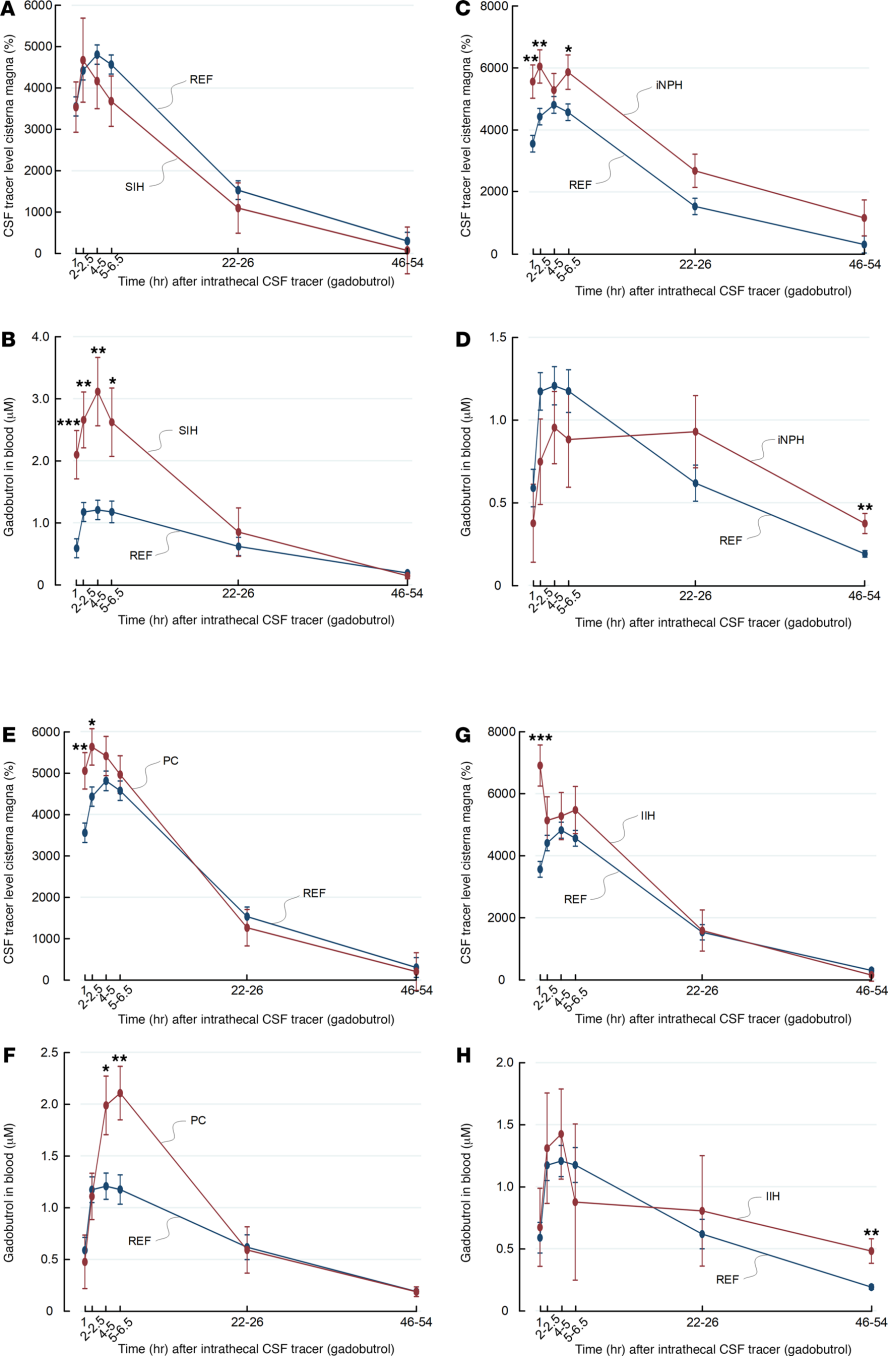
Trend plots of tracer levels in cisterna magna as assessed by MRI and in blood for different patient cohorts. Enrichment of tracer in CSF of cisterna magna is shown as trend plot of percentage change in MRI signal unit ratio for (**A**) reference cohort (REF) (blue) and subjects with spontaneous intracranial hypotension (SIH) (red), (**C**) REF (blue) and individuals with idiopathic normal pressure hydrocephalus (iNPH) (red) individuals, (**E**) REF (blue) subjects and individuals with pineal cyst (PC) (red), and (**G**) REF (blue) and individuals with IIH (red). Tracer cleared from CSF to blood is presented as concentrations of gadobutrol in whole blood for (**B**) REF and subjects with SIH, (**D**) REF individuals and patients with iNPH, (**F**) REF individuals and patients with PC, and (**H**) REF individuals and patients with IIH. Trend plots are shown as mean ± SEM, estimated from the linear-mixed model of repeated measurements. *P < 0.05, **P < 0.01, ***P < 0.001.

Figure 3 shows trend plots of tracer enrichment in CSF of cisterna magna and blood concentrations for different patient groups. In SIH patients with CSF leakage, tracer enrichment in CSF of cisterna magna was similar to that in REF subjects (Figure 3A), whereas tracer blood concentrations were significantly higher after 1, 2–2.5, 4–5, and 5–6.5 hours (Figure 3B). This indicates that CSF leakage is accompanied by high blood levels of tracer at early time points, presumably owing to pathological leakage from CSF spaces to peripheral tissue.

In this cohort of 7 patients with iNPH, CSF tracer levels in cisterna magna were significantly higher in iNPH patients up to 5–6.5 hours after intrathecal administration (Figure 3C), whereas the blood concentration of tracer was significantly higher after 48 hours (Figure 3D).

Among the 8 individuals who later underwent surgery for symptomatic nonhydrocephalic pineal cyst (PC) with subsequent improvement of symptoms, tracer enrichment in cisterna magna was higher than in REF subjects after 1 and 2–2.5 hours (Figure 3E), whereas tracer blood concentration was higher in patients with PC than in REF subjects at 4–4 and 5–6 hours (Figure 3F). For patients with IIH, CSF tracer levels in cisterna magna were comparable between patients with IIH and REF subjects (Figure 3G), except for the first time point. However, the tracer blood concentration was significantly higher after 48 hours in subjects with IIH (Figure 3H). Age-adjusted values are given for tracer enrichment in CSF of cisterna magna (Supplemental Figure 1) and blood concentrations (Supplemental Figure 2).

### Gadobutrol population pharmacokinetic modeling.

**Figure 4:**
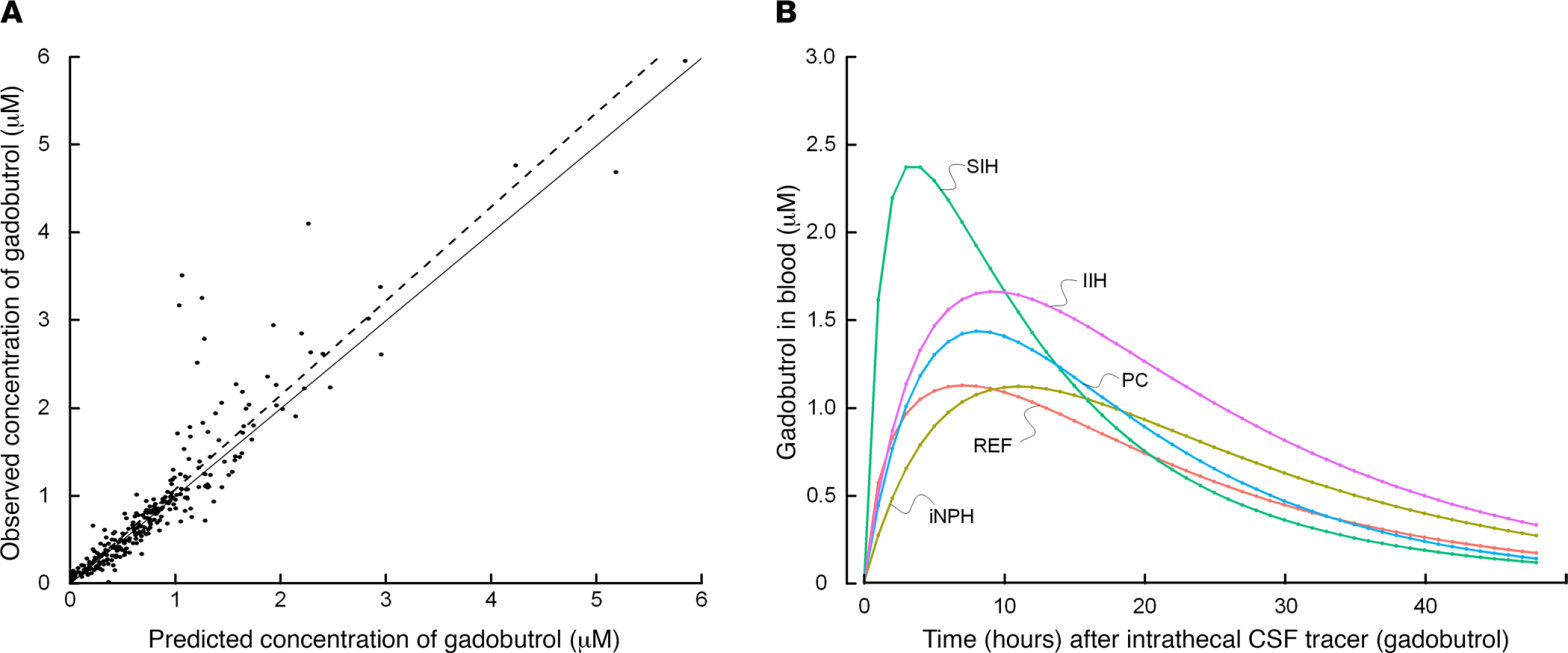
Gadobutrol population pharmacokinetic modeling. (**A**) Comparison of observed and predicted gadobutrol whole blood concentrations as described by the 2-compartment model with first-order elimination from the central compartment. (**B**) Groupwise mean gadobutrol concentration over time determined according to the 2-compartment model. Individual posterior predicted concentrations were averaged at each predicted time point from 0 to 48 hours.

We established a 2-compartment model with first-order elimination from the central compartment; the model described the data adequately (Figure 4A). The final model achieved a mean predictive error of –0.069 and root mean square error of 39.4%. The Akaike information criterion and Bayesian information criterion were 143 and 165, respectively. The model-based gadobutrol blood concentrations for the different patient groups are shown in Figure 4B, indicating different profiles for the various patient groups.

A summary of the posterior, individual pharmacokinetic parameters for gadobutrol clearance from CSF to blood is shown in Table 3. The absorption half-life (*t*_1/2,__absorption_) of gadobutrol to blood, the time taken for one-half of the administered dose to be absorbed from CSF to blood, was used to assess gadobutrol clearance from CSF to blood. In the reference group, mean *t*_1/2,__absorption_ was 6.3 ± 4.4 hours, and the maximum concentration (*C*_max_) 1.4 ± 0.6 μM was achieved after 9.8 ± 4.7 hours (Table 3).

## Discussion

The present study provides evidence that CSF tracer clearance can be measured when delivering a tracer to the CSF and thereafter determine tracer concentrations in blood at selected time points. Moreover, the time course of gadobutrol clearance from CSF to blood varies between patients with different neurological diagnoses.

### CSF efflux routes.

A detailed understanding of molecular passage routes from subarachnoid CSF to lymphatics and blood is lacking. The tracer applied in the current study distributes freely in CSF and enriches all brain regions outside blood vessels (12). Here, gadobutrol is hypothesized to serve as surrogate marker for clearance of other drugs administered in CSF as well as endogenous molecules from brain metabolism after their excretion into the interstitial and perivascular brain compartment. Clearance of neurotoxic metabolites from CSF and the brain is hampered when meningeal lymphatic clearance mechanisms are defective (13). Although molecular efflux from CSF via the cribriform plate has traditionally been considered important, this efflux route was recently reported to be of minor importance in humans (14). The cranial and spinal nerve roots are also probable efflux roots, but their relative importance remain unclear (15). The role of direct transfer of molecules from CSF to veins via arachnoid granulations remains unclear (16), whereas the parasagittal dura seems to represent a direct passage route to the meningeal lymphatic structures (17). Advanced MR imaging seems crucial for assessment of CSF distribution (18) and CSF-CNS exchange (12), but more cost-effective and easily applicable approaches would be desirable for estimation of molecular clearance from the craniospinal compartment. The blood concentration of tracer is independent of the relative role of the different efflux routes, and provides for a final output representing the overall, CSF-mediated craniospinal molecular clearance.

### Molecular clearance from CSF to systemic circulation.

So far, assessment of CSF tracer clearance has received limited attention. In a preliminary study, Verma et al. (19) reported passage of intrathecal ^99m^Tc-DPTA to urine in volumes that related to the volume of injected substance. The authors proposed that intrathecal ^99m^Tc-DPTA might be used to assess molecular exchange between the CSF and periphery in neurological diseases. A PET study using a tau PET ligand reported efflux to the nasal turbinate and reduced clearance from cerebral ventricles in patients with Alzheimer’s disease (20). It may be considered a limitation, however, that radioactive ligands are expensive and that the diagnostic process is time-consuming, which may prevent a more widespread use. Moreover, the methodology is associated with a radiation dose to the body (21), and it is questionable whether a tracer half-life of 6 hours would be long enough to sufficiently detect the differences between groups we observed at much later time points.

The literature on intrathecal opioids has previously demonstrated great variation in distribution volumes and clearance rate depending on the hydrophilic character of the opioids (22). Similarly, molecular size, relative hydrophilic, and lipophilic characters are all variables that would affect molecular CSF clearance.

### MRI contrast agents as CSF tracers.

The efflux route of molecules from CSF may differ, depending on their molecular properties. We have previously used gadobutrol and visualized tracer passage to the entire brain (12, 23) and to the parasagittal dura (17) using standardized MRI T1 sequences. However, to retrieve information about exact quantities in CSF and brain using MRI, T1 maps would have to be applied. However, in blood, bound Gd within gadobutrol can readily be detected and quantified in minute amounts down to the order of picomole. In our experience, 2.5–500 nM are feasible concentrations, and the detection threshold in this study was about 1.35 nM. In several studies, we have provided evidence that intrathecal gadobutrol in low doses (0.5 mmol or lower) is safe (24, 25), and we administered intrathecal gadobutrol in a dose that is commonly used off-label for workup of CSF leaks in the clinic. The dosage, with aim of clearance assessment, can be reduced as the exogenous element Gd in gadobutrol is stable and detectable at very low concentrations in blood. The amount of Gd measured in the blood samples was at the higher end of optimal measuring range, which implies that the dosage can be reduced. We would expect that gadobutrol passes the BBB to a very limited degree and that blood concentrations largely reflect clearance along extravascular pathways. It should be noted, however, that aging and neurodegenerative disease are accompanied with impaired BBB integrity (26, 27), and evidence of BBB disruption has been observed in both patients with IIH (28) and iNPH (29). Therefore, we hypothesize that the methodology could be extended to incorporate use of other contrast agents that are already approved for intrathecal use, including most contrast agents used in x-ray and CT exams. These contrast agents are all eliminated from blood strictly through the kidneys, allowing for measurement of tracer concentration in urine as a possible option.

### Implications of observed CSF elimination time course.

Our data indicate that gadobutrol concentrations in blood (see Figure 4) peak before peak tracer enhancement after about 24 hours in the parasagittal dura, serving as a link toward meningeal lymphatic vessels (17) and in neck lymph nodes (30). The role of cranial lymphatic clearance for gross CSF molecular clearance capacity needs to be determined. The present observations that blood concentrations peaked several hours before the previously shown tracer enrichment in parasagittal dura (17) may perhaps indicate a relatively minor role for molecular efflux over the brain convexities, and possibly a more important role of spinal efflux pathways. In line with this, recent studies suggest an important role of spinal efflux routes (31–33). With regard to molecular clearance from brain tissue, we have previously shown high association between tracer level in brain and CSF during both the enhancement and elimination phases after intrathecal administration, pointing at CSF clearance in having a decisive role for clearance of endogenous by-products from brain metabolism (12, 34). Given that CSF communicates directly with both the brain (12) and meningeal lymphatic vessels (35), it seems likely that impaired meningeal CSF clearance is the major determinate also for extravascular brain clearance.

### Estimating CSF clearance of intrathecally administered drugs.

Although it was previously thought that a substance delivered to the CSF would penetrate the brain only a few millimeters into the cortical substance (36), we provided semiquantitative evidence from intrathecal contrast-enhanced MRI that a molecule of certain properties will distribute within the entire CNS (12). Therefore, intrathecal drug administration has the advantage of direct access to the CNS as it is not restricted by the BBB as systemically administered drugs (6). For example, antisense oligonucleotides (ASOs) that are presently developed for many CNS diseases require administration to the CSF for direct access to the CNS (5, 37). The ASO drug Spinraza, delivered by intrathecal injection, was recently approved for treatment of the neurodegenerative disease spinal muscular atrophy (2, 38). Moreover, the preclinical results of intrathecal ASOs and adeno-associated viral vector–mediated gene delivery to CNS are promising in treatment for other neurodegenerative diseases such as ALS, dementia disease, and spinocerebellar ataxia (1, 39–42). Another field is neurooncology, in which intrathecal chemotherapy may be associated with dose-related neurotoxic side effects (43, 44). However, estimation of CSF clearance from CSF, as described here, has previously not been part of assessment. Hypothetically, a measure of total molecular CSF clearance capacity to blood would be useful for dose estimation of therapeutic drugs.

### Estimating CSF clearance in spontaneous intracranial hypotension.

The assessment of tracer clearance capacity from CSF to blood may have a role in various neurological diseases characterized by CSF disturbance. For example, in SIH patients it may be difficult to identify the site of CSF leakage (45). The visualization of CSF leakage has traditionally included MRI (45, 46), contrast-enhanced CT myelography (45), or intrathecal ^99m^Tc-DPTA nuclear imaging (47). As presented here, gadobutrol in blood had higher concentration in SIH than REF subjects, thereby providing in vivo evidence for CSF leakage independent of imaging. Accordingly, in patients with questionable SIH and CSF leakage, determining concentration of a tracer in blood after injection to the CSF may be applied as a screening test to assess whether leakage is present before proceeding to imaging efforts that may be heavy on resources as well as radiation.

### Craniospinal molecular clearance assessment in neurodegenerative disease.

In dementia, early diagnosis is mandatory for effective intervention. Most blood tests proposed for screening of dementia risk, or to diagnose established disease, aim to measure levels of circulating metabolic by-products such as Aβ and tau, which measures metabolic clearance indirectly (10).

Normal CNS function depends on adequate clearing of metabolic by-products. Even though the brain constitutes only about 2% of the body weight, it is the most energy-demanding organ and receives 20% of the cardiac output. Approximately 3 g of proteins are produced daily from brain metabolism, i.e., 3- to 4-fold higher protein synthesis rates than in skeletal muscle (48, 49). Days are required for clearance of proteins from the brain. Possibly, gadobutrol might be used as a surrogate marker of metabolic by-products excreted via CSF. A significant amount of Aβ isoforms are indeed cleared via CSF; about one-quarter of Aβ is cleared via CSF in rodents (50, 51). Tau to some extent passes across the BBB, particularly in the presence of BBB disruption. Hence, after injection to the cerebral ventricles, some forms of tau appeared in blood (52), and tau pathology was more severe in mice with BBB dysfunction (53). However, it seems that tau mostly does not pass across the BBB, and that a significant amount is excreted via CSF. Therefore, excretion of tau was significantly reduced in mice without dural lymphatic drainage (54), and there was a significant association between tau in blood and CSF (54). Accordingly, a test of CSF tracer clearance may be hypothesized to have better sensitivity to detect impaired clearance of endogenous neurotoxic molecules excreted along the same extravascular pathways as gadobutrol, not least in individuals with increased risk of dementia/Alzheimer’s disease or in chronic traumatic encephalopathy (55).

The estimation of CSF tracer clearance may be considered an analog to the estimation of GFR, which is the most important measure for kidney clearance function (11). GFR is accurately estimated by determining clearance of the exogenous filtration marker and x-ray contrast medium iohexol (11). In daily use, estimation of GFR is crucial for determining the safety of intravenously administered drugs. Likewise, we hypothesize the ability to accurately estimate craniospinal clearance capacity may also prove useful in predicting clearance of solutes and drugs circulating within the CSF. Here, assessment of CSF clearance using MRI contrast agent may be combined with imaging at patient level to assess the potential for distribution of drugs within brain tissue and the spinal cord, whereas in the latter, perivascular spaces have also been proven to directly communicate with the subarachnoid space (56).

### Clinical use of a test for CSF tracer clearance.

In the clinical context, assessing CSF tracer clearance may require only 1 blood sample. After intrathecal administration of the tracer, the blood sample is obtained at the desired time point, depending on clinical indication for the test. In different settings, e.g., to test for CSF leakage, the blood sample may be obtained after a few hours. When suspecting delayed clearance, e.g., in neurodegenerative disease, the blood sample may more preferably be obtained at a time point within the range of 1–2 days. We are in a process to establish reference values for age-related molecular CSF clearance over time, against which a single blood sample may be compared. In later developments, more detailed information about age-related clearance impairment needs to be established. Other physiological variables such as clearance rate from blood (GFR) may also need to be considered. Half-life of gadobutrol in blood is 1.5 hours (57).

### Limitations.

Some limitations should be noted. In this study, the amount of gadobutrol injected intrathecally was standardized to 0.5 mmol, regardless of fundamental patient characteristics such as dimensions of the craniospinal compartment, which may be a determining factor for CSF clearance capacity. Dose adjustments at individual level should be considered in future applications. The most optimal time points for retrieving blood samples could probably also be further sophisticated, both in the setting of suspected hyperaccelerated and delayed CSF clearance.

It may be considered a limitation that intrathecal gadobutrol is used off-label. In our experience, intrathecal gadobutrol in a dose of 0.5 mmol is safe (24, 25). Moreover, a recent systematic review concluded that serious adverse events have not been reported from administering intrathecal gadobutrol in a dose of 1.0 mmol or lower (58). Nevertheless, future studies should examine the utility of lower doses of gadobutrol.

The present blood concentrations of gadobutrol were measured in whole blood that was partly coagulated, which implied an extensive cleanup procedure involving homogenization of the samples. Gadobutrol is highly water soluble and eliminated from plasma through renal excretion, and it would be more convenient to analyze gadobutrol in whole blood samples with added anticoagulants or maybe in the plasma or serum fraction. Only a small amount of sample is needed (<0.5 mL) for gadobutrol analysis in blood. An optimization of the cleanup and analysis procedure may decrease the detection limit of the method even more. Bearing in mind that the amount of gadobutrol measured in the blood samples was at the higher end of optimal measuring range, this implies that the gadobutrol dosage needed for the purpose of CSF clearance assessment alone can be reduced significantly.

It also needs to be resolved to which extent assessment of gadobutrol clearance from CSF to blood is valid as a surrogate marker to predict clearance along similar pathways for neurotoxic by-products of metabolism (e.g., isoforms of Aβ and tau). In this respect, benefits of using contrast agents are several but are primarily their hydrophilic properties, stability in biological tissue, and ability to remain strictly confined to the outside of CNS blood vessels, thereby tracing extravascular CSF excretion pathways.

Finally, this study includes selected groups of patients, examined for tentative CSF disturbances of unclear cause, who we presently have in our database with whole blood samples after intrathecal contrast-enhanced MRI. The mechanisms underlying symptoms in these cases are unclear. Moreover, this is most evident for individuals with symptomatic nonhydrocephalic pineal cysts (PCs), in which some may present with pathologic intracranial pressure (ICP) indices (59) and even excellent clinical response to cyst removal (60). Since there was a clinical reason for MRI in all individuals and all individuals had symptoms, the REF cohort cannot be considered a healthy and normal cohort. However, in these individuals no apparent CSF disturbance was verified.

### Conclusions.

CSF tracer clearance assessment is clinically feasible and may provide a way to predict extravascular clearance of intrathecal drugs and endogenous metabolites from the CNS. The time course of tracer level in blood concurred well with that in CSF, which was estimated here semiquantitatively using MRI. The peak of tracer in blood preceded by far the peak of enhancement in extracranial lymphatic structures as shown in previous studies, indicating that the spinal canal has a major role in CSF clearance. Further studies are required to more precisely determine the clinical utility of the methodology and show how it differentiates different neurological diseases.

## Methods

### Patients.

Individuals who were candidates for intrathecal contrast-enhanced MRI were referred to Department of Neurosurgery, Oslo University Hospital, for tentative CSF disturbances. Patients who showed no evidence of CSF disturbance were denoted REF subjects. In these individuals, no indication for surgery was found. Patients with SIH who had a defined leakage requiring surgery to close the leakage were denoted as SIH. The diagnosis of iNPH was based on clinical, imaging findings, and results of ICP monitoring, as previously described (61, 62). Additionally, patients were included who presented a clinical improvement following shunt surgery, qualifying for the diagnosis definite iNPH, according to the Japanese guidelines (63). We also included patients with PCs who later underwent surgery with cyst removal and postoperative clinical improvement. Furthermore, individuals with IIH were included. They were subsequently shunted and reported improvement thereafter.

No patients with a history of hypersensitivity reactions to contrast agents, severe allergy reactions in general, or evidence of renal dysfunction were included. We also did not include pregnant or breastfeeding women or individuals under 18 or more than 80 years of age.

### Intrathecal tracer administration.

**Figure 5:**
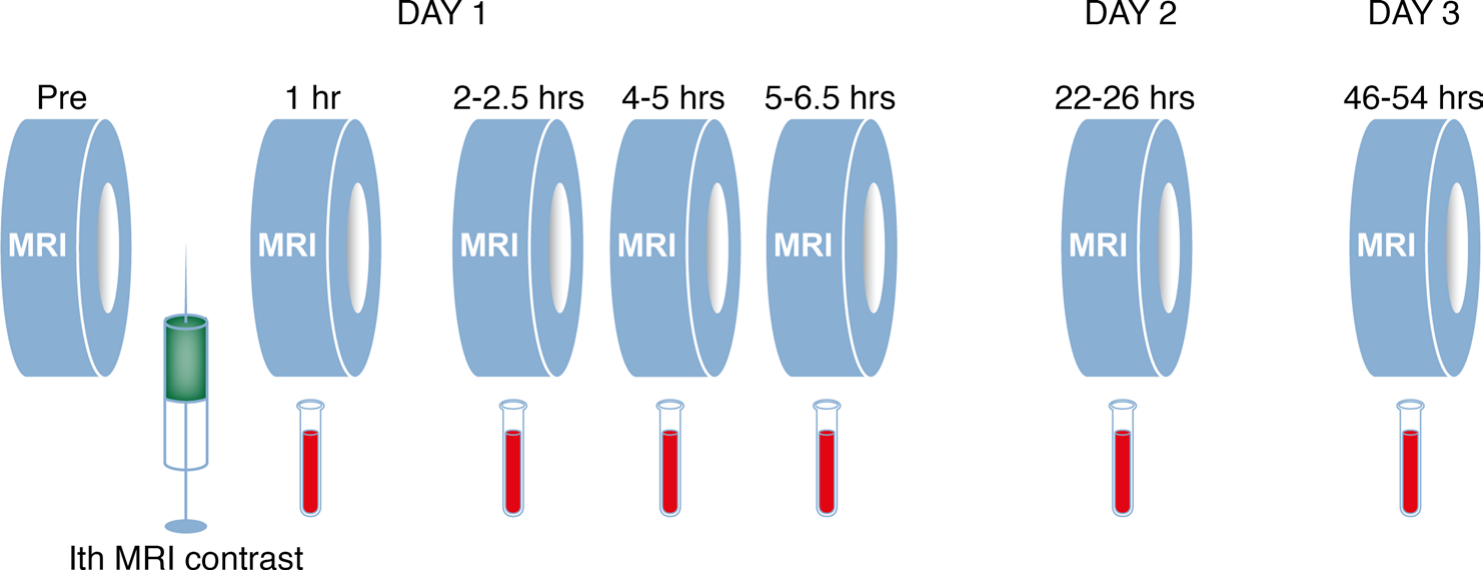
Study design. A standardized protocol was followed in all study participants. T1-weighted MRI scanning was performed before the intrathecal (Ith) MRI contrast agent injection (Pre), and thereafter standardized T1-weighted MRI sequences were obtained at defined time points at 1, 2–2.5, 4–5, 5–6.5, 22–26, and 46–54 hours. Blood samples for whole blood concentration estimation of MRI contrast agent were obtained in conjunction with each of the MRI acquisitions. Illustration: Øystein Horgmo, University of Oslo.

The intrathecal injection was performed by an interventional neuroradiologist. Correct position of the spinal needle in the subarachnoid space was verified by CSF backflow from the puncture needle, and injecting of a small amount (typically 3 mL) of 270 mg L/mL iodixanol (Visipaque, GE Healthcare) confirmed unrestricted distribution of radiopaque contrast agent in the lumbar subarachnoid space. The MRI contrast agent gadobutrol (Gadovist, Bayer Pharma AG) was given intrathecal in a volume of 0.5 mL (1.0 mmol/mL) at a speed of a few seconds. The study design with repeated MRI acquisitions and blood samples is presented in Figure 5.

### MRI acquisitions.

A 3 Tesla Philips Ingenia MRI scanner (Philips Medical Systems) was used, with equal imaging protocol settings at each acquisition to obtain sagittal 3D T1-weighted volume scans. The imaging parameters included: repetition time = “shortest” (typically 5.1 ms), echo time = “shortest” (typically 2.3 ms), Flip angle = 8 degrees, field of view = 256 × 256 cm and matrix = 256 × 256 pixels (reconstructed 512 × 512), and slice thickness 1 mm. The MRI exams were categorized as follows: 1, 2–2.5, 4–5, 5–6.5, 22–26, and 46–54 hours. Tracer enrichment in CSF of cisterna magna was assessed by placing region of interest within the CSF (Supplemental Figure 2). To correct for baseline changes in image gray scale, a reference ROI was placed in the superior sagittal sinus in axially reconstructed images from the same T1 volume scan. The ratio refers to as normalized T1 signal units and percentage change in normalized T1 signal units from before contrast was determined. We have verified that the T1 signal within the sagittal sinus/confluence did not increase following intrathecal gadobutrol administration (Supplemental Figure 3).

### Chemical analysis of Gd in blood.

Intravenous blood samples were obtained and kept in a freezer at –80°C. The blood samples were thawed and transferred to a 15 mL polyethylene vial and homogenized with an Ultra-Turrax homogenizer (IKA T18). Blanks were regularly prepared with a similar procedure with 6 mL ultrapure deionized Milli-Q water to control for background contamination from the Ultra-Turrax. Aliquots of the homogenized blood samples (approximately 0.6 g) were then subjected to microwave-assisted digestion with concentrated ultrapure distilled nitric acid mixed with ultrapure deionized Milli-Q water (3 mL water and 5 mL nitric acid). The samples were digested in an UltraCLAVE single reaction chamber microwave oven (Milestone) according to a 60-minute stepwise heating program, where hold time for maximum temperature (250°C) was 15 minutes. After digestion, the samples were allowed to cool down to room temperature into their vessels before being transferred to 50 mL polyethylene vials (VWR) and diluted with deionized ultrapure water. Two certified plant reference material from the National Institute of Standards & Technology (NIST), 1515 apple leaves and 1547 peach leaves containing 3 and 1 μg/g Gd, respectively, were subjected to similar microwave-assisted digestion to assess the recovery of Gd. Mean recoveries of Gd in the NIST apple leaves and NIST peach leaves were 95% ± 4.1% SD and 103% ± 7.7% SD, respectively. The blood samples were analyzed for Gd by inductively coupled plasma mass spectrometry (Agilent 7700x, single quadrupole MS; Agilent Technologies). Indium at 0.1 μg/L was used as internal standard. The samples were quantified using a 5-point standard curve (0.01–10 μg/L), and results were corrected for blank values. Representative detection limits for the analytics, estimated as 3 times the SD of 20 method blanks from 5 analytical runs, was 0.0009 μg/L. To ensure the correct quantification of the metals, 2 reference solutions of known element concentrations (0.1 and 1.0 μg/L) were analyzed in addition to in-house prepared standards. A deviation of 9% from the given concentration in the reference solution was accepted. Blanks were regularly analyzed to control for background contamination. The measured Gd concentrations in the blood samples were recalculated to gadobutrol concentrations.

### Gadobutrol population pharmacokinetic modeling.

To accurately describe individual tracer (gadobutrol) pharmacokinetics, a population pharmacokinetic model was developed. A nonparametric adaptive grid approach implemented in Pmetrics (version 1.9.7) for R (version 4.0.0) was used (64). A total of 277 whole blood samples were included in the complete data set, across 52 individuals. Covariates were not included, owing to sole interest in individual pharmacokinetic predictions.

Posterior individual parameter values as well as posterior individually predicted concentrations obtained from the final population pharmacokinetic model were used for all pharmacokinetic calculations. The *t*_1/2,__absorption_ (time to 50% of tracer dose absorbed from CSF to blood) was used as a measure of molecular CSF clearance to blood, and determined by dividing the natural logarithm of 2 by the model estimated transfer rate constant from CSF to the central compartment. Area under curve from 0 to infinity (AUC_0-¥_) was calculated with the trapezoidal approximation from the individual posterior predicted concentrations using the “makeAUC”-function” in Pmetrics.

### Statistics.

Repeated measurements were assessed with linear-mixed models using a subject-specific random intercept with an independent structure of residual errors at 1–24 and at 48 hours (i.e., assuming a different residual error at 48 hours compared with the previous observation times) using maximum likelihood estimation. We tested the difference between the groups at the different times after intrathecal CSF tracer with pairwise comparisons. The model-based parameters were compared between groups, using independent *t* test samples (2 tailed). For statistical analysis, we applied SPSS version 26 (IBM Corporation) or Stata/SE 15.0 (StataCorp LLC). A *P* value of 0.05 was considered significant.

### Study approval.

The study was approved by the Regional Committee for Medical and Health Research Ethics of Health Region South-East, Oslo, Norway (2015/96); the IRB of Oslo University Hospital (2015/1868); and the National Medicines Agency, Oslo, Norway (15/04932-7). Participants of this prospective and observational study were included after written and oral informed consent were provided from January 2017 to May 2018.

## Author contributions

PKE and GR conceptualized and designed the project. PKE and AL handled the blood samples. EM and HU performed the blood analysis. HC and MHH created the pharmacokinetic model. AHP, MHH, and PKE performed the statistical analysis. PKE and GR supervised and performed the administration and writing of the original draft. PKE, EM, UH, AL, AHP, BH, and GR wrote, reviewed, and edited the manuscript. All authors approved the final version of the manuscript.

## Supplementary Material

Supplemental data

Trial reporting checklists

ICMJE disclosure forms

## Figures and Tables

**Table 1:**
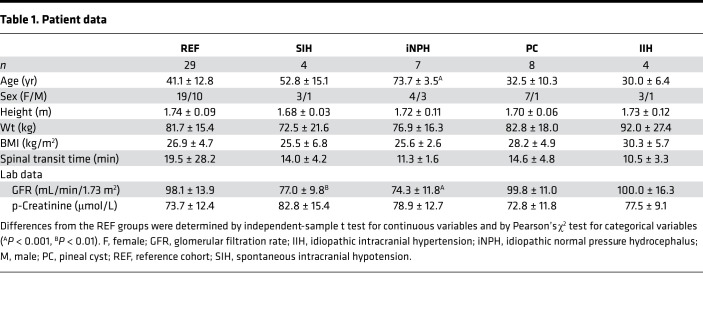
Patient data

**Table 2:**
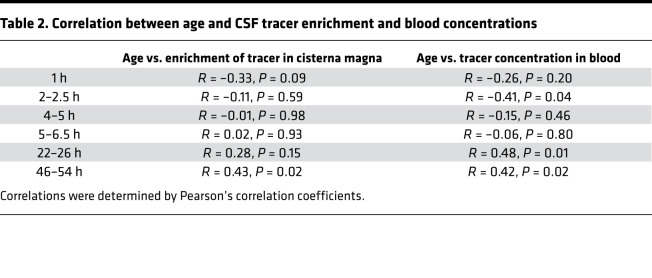
Correlation between age and CSF tracer enrichment and blood concentrations

**Table 3:**
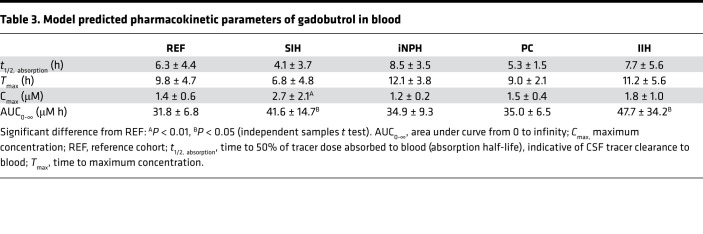
Model predicted pharmacokinetic parameters of gadobutrol in blood

## Version 1 04/06/2021

In-Press Preview

## Version 2 05/10/2021

Electronic publication
