# Supplementary material for: Clinical application of intrathecal gadobutrol for assessment of cerebrospinal fluid tracer clearance to blood
Source: JCI Insight. 2021 May 10;6(9):e147063. doi: 10.1172/jci.insight.147063 (PMC8262318; doi:10.1172/jci.insight.147063)
Supplement: Supplemental data [file jciinsight-6-147063-s206.pdf]

Clinical application of intrathecal gadobutrol for assessment of cerebrospinal fluid tracer clearance to blood

Per Kristian Eide[1,2*] Espen Mariussen[3], Hilde Uggerud[3], Are H. Pripp[4], Aslan Lashkarivand[1,2], Bjørnar Hassel[2,5], Hege Christensen[6], Markus Herberg Hovd[6], Geir Ringstad[7]

[1]Department of Neurosurgery, Oslo University Hospital – Rikshospitalet, Oslo, Norway.
[2]Institute of Clinical Medicine, Faculty of Medicine, University of Oslo, Oslo, Norway.
[3]Norwegian Insitute for Air Research, Kjeller, Norway.
[4]Oslo Centre of Biostatistics and Epidemiology, Research Support Services, Oslo University Hospital, Oslo, Norway.
[5]Department of Neurohabilitation, Oslo University Hospital, Oslo, Norway
[6]Section for Pharmacology and Pharmaceutical Biosciences, Department of Pharmacy, University of Oslo, Oslo, Norway[7]Division of Radiology and Nuclear Medicine, Department of Radiology, Oslo University Hospital - Rikshospitalet, Oslo, Norway.

*Correspondence:

Professor Per Kristian Eide, MD PhD
Department of Neurosurgery
Oslo University Hospital - Rikshospitalet
Pb 4950 Nydalen,

N-0424 Oslo, Norway

p.k.eide@medisin.uio.no/peide@ous-hf.no

Supplementary Figure 1. Enrichment of tracer within cisterna magna

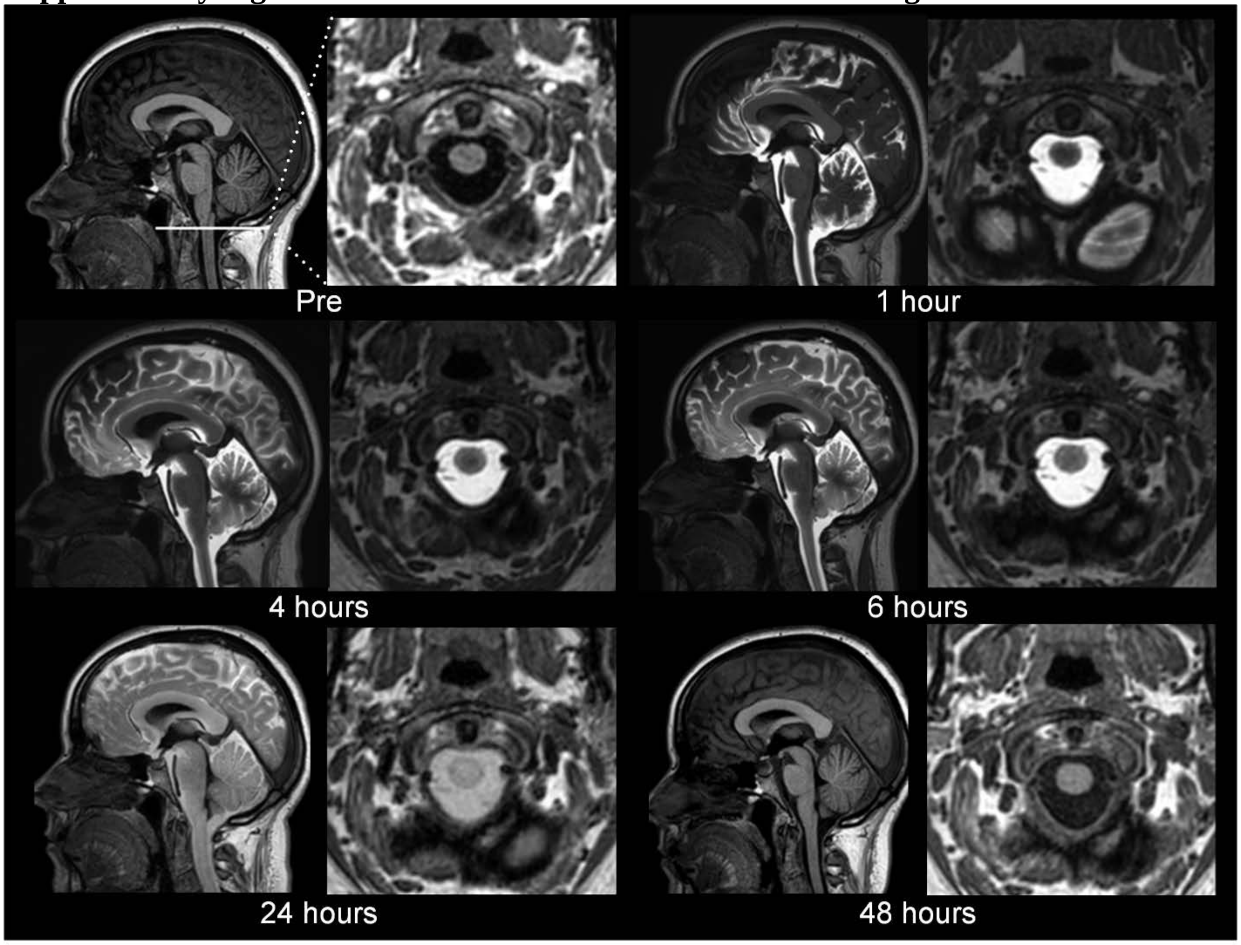

The change in MRI T1 signal within the cisterna magna is shown before (Pre) and 1, 4, 6, 24 and 48 hours after injection of intrathecal gadobutrol in one REF subject

Supplementary Figure 2. Placement of region of interest within cisterna magna

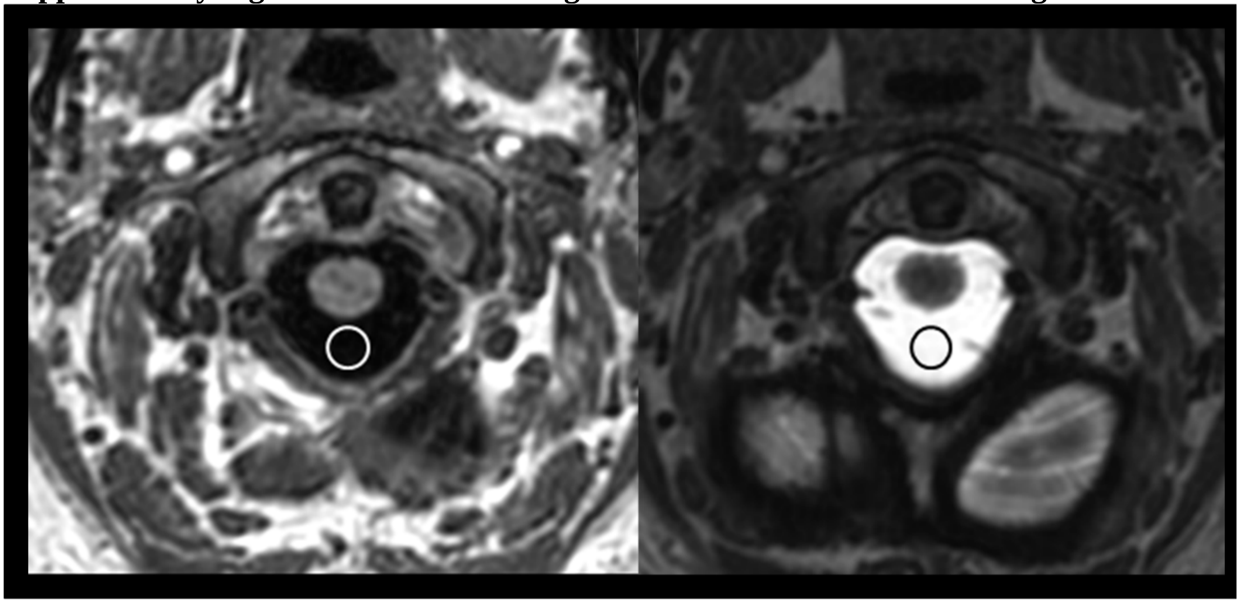

The placement of region of interest (open circle) within the cisterna magna is shown before (left) and 1 after (right) injection of intrathecal gadobutrol.

Supplementary Figure 3. Change in MRI T1 within sagittal sinus/confluence over time

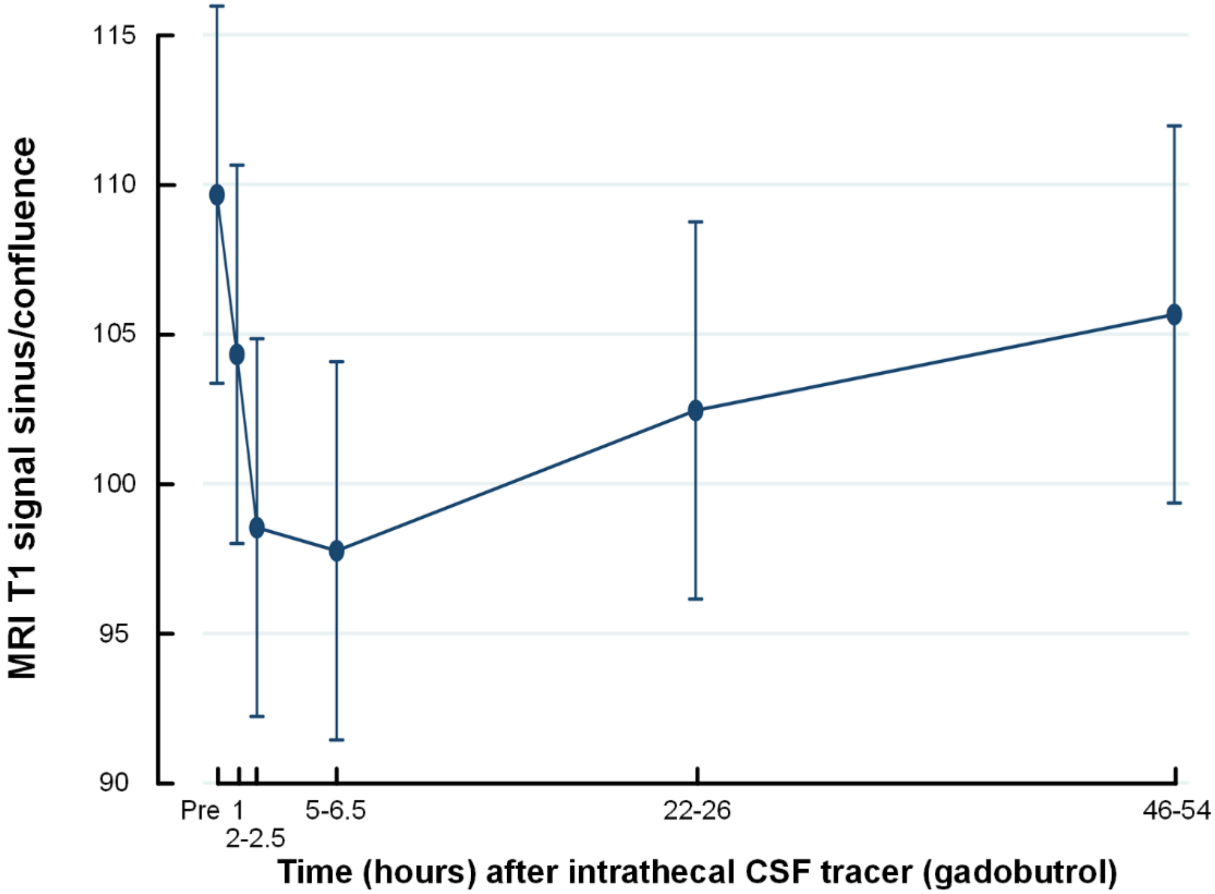

The change in mean MRI T1 signal within the sagittal sinus/confluence before (Pre) and 1, 2-2.5, 5-6.5, 22-26 and 46-54 after injection of intrathecal gadobutrol. Bars represented by standard error. Provided gadobutrol enriched blood, a T1 signal increase would be expected, which is not seen. The trend plot does not indicate enrichment of tracer within the sagittal sinus/confluence.

Suppl Table 1. Age-adjusted differences in CSF tracer enrichment between REF patients and patients with CSF disturbances

	1 hr		2-2.5 hrs		4-5 hrs		5-6.5 hrs		22-26 hrs		46-54 hrs	
	**Mean±SE**	**P-value**	**Mean±SE**	**P-value**	**Mean±SE**	**P-value**	**Mean±SE**	**P-value**	**Mean±SE**	**P-value**	**Mean±SE**	**P-value**
Diff SIH - REF	-16 ± 651	0.980	250 ± 1042	0.810	-640 ± 707	0.366	-886 ± 651	0.174	-433 ± 650	0.505	-227 ± 602	0.706
Age-adjusted Diff SIH - REF	-37 ± 667	0.956	232 ± 1050	0.825	-662 ± 725	0.362	-906 ± 668	0.175	-453 ± 666	0.496	-248 ± 619	0.689
P-value of age		0.889		0.889		0.889		0.889		0.889		0.889
Diff iNPH - REF	2005 ± 600	0.001	1620 ± 599	0.007	477 ± 601	0.428	1294 ± 617	0.036	1150 ± 598	0.055	858 ± 641	0.181
Age-adjusted Diff iNPH - REF	1906 ± 827	0.021	1520 ± 827	0.066	377 ± 827	0.649	1193 ± 842	0.156	1050 ± 826	0.204	758 ± 858	0.377
P-value of age		0.861		0.861		0.861		0.861		0.861		0.861
Diff PC - REF	1496 ± 499	0.003	1201 ± 498	0.016	600 ± 530	0.257	390 ± 512	0.446	-269 ± 497	0.589	-99 ± 514	0.848
Age-adjusted Diff PC - REF	1399 ± 510	0.006	1105 ± 509	0.030	505 ± 540	0.350	294 ± 523	0.573	-365 ± 508	0.472	-195 ± 526	0.711
P-value of age		0.415		0.415		0.415		0.415		0.415		0.415
Diff IIH - REF	3346 ± 710	0.001	730 ± 801	0.362	454 ± 804	0.572	911 ± 802	0.256	57 ± 707	0.936	-149 ± 208	0.472
*Age-adjusted Diff IIH - REF	-	-	-	-	-	-	-	-	-	-	-	-
P-value of age												

*Age-adjusted differences between IIH and REF subjects could not be determined due to low number of observations.

Suppl Table 2. Age-adjusted differences in blood concentrations of CSF tracer between REF patients and patients with CSF disturbances

	**1 hr**		**2-2.5 hrs**		**4-5 hrs**		**5-6.5 hrs**		**22-26 hrs**		**46-54 hrs**	
	**Mean±SE**	**P-value**	**Mean±SE**	**P-value**	**Mean±SE**	**P-value**	**Mean±SE**	**P-value**	**Mean±SE**	**P-value**	**Mean±SE**	**P-value**
Diff SIH - REF	1.51 ± 0.42	<0.001	1.48 ± 0.48	0.002	1.91 ± 0.57	0.001	1.45 ± 0.58	0.012	0.23 ± 0.42	0.58	-0.05 ± 0.05	0.37
Age-adjusted Diff SIH - REF	1.47 ± 0.42	<0.001	1.44 ± 0.48	0.003	1.86 ± 0.58	0.001	1.45 ± 0.58	0.013	0.197 ± 0.418	0.64	-0.08 ± 0.05	0.09
P-value of age		0.008		0.008		0.008		0.008		0.008		0.008
Diff iNPH - REF	-0.21 ± 0.26	0.416	-0.43 ± 0.28	0.132	-0.25 ± 0.25	0.306	-0.29 ± 0.32	0.36	0.31 ± 0.24	0.202	0.18 ± 0.06	0.004
Age-adjusted Diff iNPH - REF	-0.29 ± 0.27	0.27	-0.50 ± 0.29	0.08	-0.33 ± 0.25	0.191	-0.37 ± 0.32	0.25	0.23 ± 0.25	0.36	0.10 ± 0.07	0.15
P-value of age		0.053		0.053		0.053		0.053		0.053		0.053
Diff PC - REF	-0.11 ± 0.29	0.70	-0.06 ± 0.26	0.80	0.78 ± 0.31	0.012	0.93 ± 0.30	0.002	-0.03 ± 0.25	0.92	-0.002 ± 0.05	0.97
Age-adjusted Diff PC - REF	-0.09 ± 0.29	0.76	-0.03 ± 0.26	0.90	0.80 ± 0.31	0.010	0.96 ± 0.30	0.001	0.002 ± 0.26	0.93	0.014 ± 0.045	0.76
P-value of age		0.009		0.009		0.009		0.009		0.009		0.009
Diff IIH - REF	0.08 ± 0.34	0.80	0.14 ± 0.46	0.77	0.22 ± 0.39	0.572	-0.30 ± 0.65	0.64	0.19 ± 0.46	0.69	0.29 ± 0.10	0.004
Age-adjusted Diff IIH - REF	0.12 ± 0.34	0.739	0.16 ± 0.47	0.733	0.25 ± 0.39	0.522	-0.29 ± 0.65	0.656	0.21 ± 0.47	0.656	0.32 ± 0.09	<0.001
P-value of age		0.029		0.029		0.029		0.029		0.029		0.029
