# Supplementary material for: Clinical application of intrathecal gadobutrol for assessment of cerebrospinal fluid tracer clearance to blood
Source: JCI Insight. 2021 May 10;6(9):e147063. doi: 10.1172/jci.insight.147063 (PMC8262318; doi:10.1172/jci.insight.147063)
Supplement: ICMJE disclosure forms [file jciinsight-6-147063-s208.pdf]

# ICMJE DISCLOSURE FORM

**Date: February 19th 2021**
**Your Name: Per Kristian Eide**
**Manuscript Title:** Clinical application of intrathecal gadobutrol for assessment of cerebrospinal fluid tracer clearance to blood
**Manuscript number (if known):** 147063-INS-CMED-TR-2

**In the interest of transparency, we ask you to disclose all relationships/activities/interests listed below that are related to the content of your manuscript. "Related" means any relation with for-profit or not-for-profit third parties whose interests may be affected by the content of the manuscript. Disclosure represents a commitment to transparency and does not necessarily indicate a bias. If you are in doubt about whether to list a relationship/activity/interest, it is preferable that you do so.**

**The following questions apply to the author's relationships/activities/interests as they relate to the current manuscript only.**

**The author's relationships/activities/interests should be defined broadly. For example, if your manuscript pertains to the epidemiology of hypertension, you should declare all relationships with manufacturers of antihypertensive medication, even if that medication is not mentioned in the manuscript.**

**In item #1 below, report all support for the work reported in this manuscript without time limit. For all other items, the time frame for disclosure is the past 36 months.**

| | | **Name all entities with whom you have this relationship or indicate none (add rows as needed)** | **Specifications/Comments (e.g., if payments were made to you or to your institution)** |
|---|---|---|---|
| | | **Time frame: Since the initial planning of the work** | |
| 1 | All support for the present manuscript (e.g., funding, provision of study materials, medical writing, article processing charges, etc.) **No time limit for this item.** | Department of Neurosurgery, Oslo University Hospital-Rikshospitalet, Oslo, Norway | |
| | | University of Oslo | Blood Gd analysis |
| | | Norwegian Institute for Air Research, Kjeller, Norway | Blood Gd analysis |
| | | **Time frame: past 36 months** | |
| 2 | Grants or contracts from any entity (if not indicated in item #1 above). | None | |
| | | | |
| | | | |
| 3 | Royalties or licenses | None | |
| | | | |
| | | | |
| 4 | Consulting fees | None | |
| | | | |

| | | | |
|---|---|---|---|
| 5 | Payment or honoraria for lectures, presentations, speakers bureaus, manuscript writing or educational events | None | |
| | | | |
| | | | |
| 6 | Payment for expert testimony | None | |
| | | | |
| | | | |
| 7 | Support for attending meetings and/or travel | None | |
| | | | |
| | | | |
| 8 | Patents planned, issued or pending | US Patent Pending | US Patent App. 16/577,227, 2020 |
| | | | |
| | | | |
| 9 | Participation on a Data Safety Monitoring Board or Advisory Board | None | |
| | | | |
| | | | |
| 10 | Leadership or fiduciary role in other board, society, committee or advocacy group, paid or unpaid | X | Board member of BrainWideSolutions AS |
| | | | |
| | | | |
| 11 | Stock or stock options | X | Shareholder in BrainWideSolutions AS |
| | | | |
| | | | |
| 12 | Receipt of equipment, materials, drugs, medical writing, gifts or other services | None | |
| | | | |
| | | | |
| 13 | Other financial or non-financial interests | None | |
| | | | |
| | | | |

**Please place an "X" next to the following statement to indicate your agreement:**

**X I certify that I have answered every question and have not altered the wording of any of the questions on this form.**

**ICMJE DISCLOSURE FORM**

**Date:**____________**19/2-2021**______________________________________________________________
**Your Name:**_______**Espen Mariussen**_______________________________________________________________
**Manuscript Title:** Clinical application of intrathecal gadobutrol for assessment of cerebrospinal fluid tracer clearance to blood
**Manuscript number (if known):** 147063-INS-CMED-TR-2

**In the interest of transparency, we ask you to disclose all relationships/activities/interests listed below that are related to the content of your manuscript. "Related" means any relation with for-profit or not-for-profit third parties whose interests may be affected by the content of the manuscript. Disclosure represents a commitment to transparency and does not necessarily indicate a bias. If you are in doubt about whether to list a relationship/activity/interest, it is preferable that you do so.**

**The following questions apply to the author's relationships/activities/interests as they relate to the current manuscript only.**

**The author's relationships/activities/interests should be defined broadly. For example, if your manuscript pertains to the epidemiology of hypertension, you should declare all relationships with manufacturers of antihypertensive medication, even if that medication is not mentioned in the manuscript.**

**In item #1 below, report all support for the work reported in this manuscript without time limit. For all other items, the time frame for disclosure is the past 36 months.**

| | | **Name all entities with whom you have this relationship or indicate none (add rows as needed)** | **Specifications/Comments (e.g., if payments were made to you or to your institution)** |
|---|---|---|---|
| | | Time frame: Since the initial planning of the work | |
| 1 | All support for the present manuscript (e.g., funding, provision of study materials, medical writing, article processing charges, etc.) **No time limit for this item.** | __x__None | |
| | | | |
| | | | |
| | | | |
| | | | |
| | | | |
| | | | |
| | | Time frame: past 36 months | |
| 2 | Grants or contracts from any entity (if not indicated in item #1 above). | __x__None | |
| | | | |
| | | | |
| 3 | Royalties or licenses | __x__None | |
| | | | |
| | | | |
| 4 | Consulting fees | __x__None | |
| | | | |

| | | | |
|---|---|---|---|
| 5 | Payment or honoraria for lectures, presentations, speakers bureaus, manuscript writing or educational events | __x__None | |
| 6 | Payment for expert testimony | __x__None | |
| 7 | Support for attending meetings and/or travel | __x__None | |
| 8 | Patents planned, issued or pending | __x__None | |
| 9 | Participation on a Data Safety Monitoring Board or Advisory Board | __x__None | |
| 10 | Leadership or fiduciary role in other board, society, committee or advocacy group, paid or unpaid | __x__None | |
| 11 | Stock or stock options | __x__None | |
| 12 | Receipt of equipment, materials, drugs, medical writing, gifts or other services | __x__None | |
| 13 | Other financial or non-financial interests | __x__None | |

**Please place an "X" next to the following statement to indicate your agreement:**

___ **I certify that I have answered every question and have not altered the wording of any of the questions on this form.**

19/2-21 Espen Marinsson

# ICMJE DISCLOSURE FORM

**Date:** **19th February 2021**
**Your Name: Hilde Thelle Uggerud**
**Manuscript Title:** Clinical application of intrathecal gadobutrol for assessment of cerebrospinal fluid tracer clearance to blood
**Manuscript number (if known):** 147063-INS-CMED-TR-2

**In the interest of transparency, we ask you to disclose all relationships/activities/interests listed below that are related to the content of your manuscript. "Related" means any relation with for-profit or not-for-profit third parties whose interests may be affected by the content of the manuscript. Disclosure represents a commitment to transparency and does not necessarily indicate a bias. If you are in doubt about whether to list a relationship/activity/interest, it is preferable that you do so.**

**The following questions apply to the author's relationships/activities/interests as they relate to the current manuscript only.**

**The author's relationships/activities/interests should be defined broadly. For example, if your manuscript pertains to the epidemiology of hypertension, you should declare all relationships with manufacturers of antihypertensive medication, even if that medication is not mentioned in the manuscript.**

**In item #1 below, report all support for the work reported in this manuscript without time limit. For all other items, the time frame for disclosure is the past 36 months.**

| | | **Name all entities with whom you have this relationship or indicate none (add rows as needed)** | **Specifications/Comments (e.g., if payments were made to you or to your institution)** |
|---|---|---|---|
| | | **Time frame: Since the initial planning of the work** | |
| 1 | All support for the present manuscript (e.g., funding, provision of study materials, medical writing, article processing charges, etc.) **No time limit for this item.** | _x__None | |
| | | | |
| | | | |
| | | | |
| | | | |
| | | | |
| | | | |
| | | | |
| | | **Time frame: past 36 months** | |
| 2 | Grants or contracts from any entity (if not indicated in item #1 above). | _x__None | |
| | | | |
| | | | |
| 3 | Royalties or licenses | __x_None | |
| | | | |
| | | | |
| 4 | Consulting fees | _x__None | |
| | | | |

| | | | |
|---|---|---|---|
| | | | |
| 5 | Payment or honoraria for lectures, presentations, speakers bureaus, manuscript writing or educational events | _x__None | |
| | | | |
| | | | |
| 6 | Payment for expert testimony | __x_None | |
| | | | |
| | | | |
| 7 | Support for attending meetings and/or travel | _x__None | |
| | | | |
| | | | |
| 8 | Patents planned, issued or pending | _x__None | |
| | | | |
| | | | |
| 9 | Participation on a Data Safety Monitoring Board or Advisory Board | __x_None | |
| | | | |
| | | | |
| 10 | Leadership or fiduciary role in other board, society, committee or advocacy group, paid or unpaid | _x__None | |
| | | | |
| | | | |
| 11 | Stock or stock options | __x_None | |
| | | | |
| | | | |
| 12 | Receipt of equipment, materials, drugs, medical writing, gifts or other services | _x__None | |
| | | | |
| | | | |
| 13 | Other financial or non-financial interests | _x_None | |
| | | | |
| | | | |

**Please place an "X" next to the following statement to indicate your agreement:**

**X I certify that I have answered every question and have not altered the wording of any of the questions on this form. X**

# ICMJE DISCLOSURE FORM

**Date:_19 February 2021__________________________________________________________________**
**Your Name:_Are Hugo Pripp_______________________________________________________________**
**Manuscript Title:** Clinical application of intrathecal gadobutrol for assessment of cerebrospinal fluid tracer clearance to blood
**Manuscript number (if known):** 147063-INS-CMED-TR-2

**In the interest of transparency, we ask you to disclose all relationships/activities/interests listed below that are related to the content of your manuscript. "Related" means any relation with for-profit or not-for-profit third parties whose interests may be affected by the content of the manuscript. Disclosure represents a commitment to transparency and does not necessarily indicate a bias. If you are in doubt about whether to list a relationship/activity/interest, it is preferable that you do so.**

**The following questions apply to the author's relationships/activities/interests as they relate to the current manuscript only.**

**The author's relationships/activities/interests should be defined broadly. For example, if your manuscript pertains to the epidemiology of hypertension, you should declare all relationships with manufacturers of antihypertensive medication, even if that medication is not mentioned in the manuscript.**

**In item #1 below, report all support for the work reported in this manuscript without time limit. For all other items, the time frame for disclosure is the past 36 months.**

| | | **Name all entities with whom you have this relationship or indicate none (add rows as needed)** | **Specifications/Comments (e.g., if payments were made to you or to your institution)** |
|---|---|---|---|
| | | **Time frame: Since the initial planning of the work** | |
| 1 | All support for the present manuscript (e.g., funding, provision of study materials, medical writing, article processing charges, etc.) **No time limit for this item.** | ____None | |
| | | | |
| | | | |
| | | | |
| | | | |
| | | | |
| | | | |
| | | | |
| | | **Time frame: past 36 months** | |
| 2 | Grants or contracts from any entity (if not indicated in item #1 above). | ____None | |
| | | | |
| | | | |
| 3 | Royalties or licenses | ____None | |
| | | | |
| | | | |
| 4 | Consulting fees | ____None | |
| | | | |

| | | | |
|---|---|---|---|
| 5 | Payment or honoraria for lectures, presentations, speakers bureaus, manuscript writing or educational events | ____None | |
| | | | |
| | | | |
| 6 | Payment for expert testimony | ____None | |
| | | | |
| | | | |
| 7 | Support for attending meetings and/or travel | ____None | |
| | | | |
| | | | |
| 8 | Patents planned, issued or pending | ____None | |
| | | | |
| | | | |
| 9 | Participation on a Data Safety Monitoring Board or Advisory Board | ____None | |
| | | | |
| | | | |
| 10 | Leadership or fiduciary role in other board, society, committee or advocacy group, paid or unpaid | ____None | |
| | | | |
| | | | |
| 11 | Stock or stock options | ____None | |
| | | | |
| | | | |
| 12 | Receipt of equipment, materials, drugs, medical writing, gifts or other services | ____None | |
| | | | |
| | | | |
| 13 | Other financial or non-financial interests | ____None | |
| | | | |
| | | | |

**Please place an "X" next to the following statement to indicate your agreement:**

**_X_ I certify that I have answered every question and have not altered the wording of any of the questions on this form.**

## ICMJE DISCLOSURE FORM

**Date: 19.02.21**_________________________________________________________________________________
**Your Name: Aslan Lashkarivand**___________________________________________________________________________
**Manuscript Title:** Clinical application of intrathecal gadobutrol for assessment of cerebrospinal fluid tracer clearance to blood
**Manuscript number (if known):** 147063-INS-CMED-TR-2

**In the interest of transparency, we ask you to disclose all relationships/activities/interests listed below that are related to the content of your manuscript. "Related" means any relation with for-profit or not-for-profit third parties whose interests may be affected by the content of the manuscript. Disclosure represents a commitment to transparency and does not necessarily indicate a bias. If you are in doubt about whether to list a relationship/activity/interest, it is preferable that you do so.**

**The following questions apply to the author's relationships/activities/interests as they relate to the current manuscript only.**

**The author's relationships/activities/interests should be defined broadly. For example, if your manuscript pertains to the epidemiology of hypertension, you should declare all relationships with manufacturers of antihypertensive medication, even if that medication is not mentioned in the manuscript.**

**In item #1 below, report all support for the work reported in this manuscript without time limit. For all other items, the time frame for disclosure is the past 36 months.**

| | | **Name all entities with whom you have this relationship or indicate none (add rows as needed)** | **Specifications/Comments (e.g., if payments were made to you or to your institution)** |
|---|---|---|---|
| | | **Time frame: Since the initial planning of the work** | |
| 1 | All support for the present manuscript (e.g., funding, provision of study materials, medical writing, article processing charges, etc.) **No time limit for this item.** | _X___None | |
| | | | |
| | | | |
| | | | |
| | | | |
| | | | |
| | | | |
| | | | |
| | | **Time frame: past 36 months** | |
| 2 | Grants or contracts from any entity (if not indicated in item #1 above). | _X___None | |
| | | | |
| | | | |
| 3 | Royalties or licenses | _X___None | |
| | | | |
| | | | |
| 4 | Consulting fees | _X___None | |
| | | | |

| | | | |
|---|---|---|---|
| 5 | Payment or honoraria for lectures, presentations, speakers bureaus, manuscript writing or educational events | _X___None | |
| | | | |
| | | | |
| 6 | Payment for expert testimony | _X___None | |
| | | | |
| | | | |
| 7 | Support for attending meetings and/or travel | _X___None | |
| | | | |
| | | | |
| 8 | Patents planned, issued or pending | _X___None | |
| | | | |
| | | | |
| 9 | Participation on a Data Safety Monitoring Board or Advisory Board | _X___None | |
| | | | |
| | | | |
| 10 | Leadership or fiduciary role in other board, society, committee or advocacy group, paid or unpaid | _X___None | |
| | | | |
| | | | |
| 11 | Stock or stock options | _X___None | |
| | | | |
| | | | |
| 12 | Receipt of equipment, materials, drugs, medical writing, gifts or other services | _X___None | |
| | | | |
| | | | |
| 13 | Other financial or non-financial interests | _X___None | |
| | | | |
| | | | |

**Please place an "X" next to the following statement to indicate your agreement:**

**_X__ I certify that I have answered every question and have not altered the wording of any of the questions on this form.**

## ICMJE DISCLOSURE FORM

**Date:**_20 Feb 2021______________________________________________________________________________
**Your Name: Bjørnar Hassel**_____________________________________________________________________
**Manuscript Title:** Clinical application of intrathecal gadobutrol for assessment of cerebrospinal fluid tracer clearance to blood
**Manuscript number (if known):** 147063-INS-CMED-TR-2

**In the interest of transparency, we ask you to disclose all relationships/activities/interests listed below that are related to the content of your manuscript. "Related" means any relation with for-profit or not-for-profit third parties whose interests may be affected by the content of the manuscript. Disclosure represents a commitment to transparency and does not necessarily indicate a bias. If you are in doubt about whether to list a relationship/activity/interest, it is preferable that you do so.**

**The following questions apply to the author's relationships/activities/interests as they relate to the current manuscript only.**

**The author's relationships/activities/interests should be defined broadly. For example, if your manuscript pertains to the epidemiology of hypertension, you should declare all relationships with manufacturers of antihypertensive medication, even if that medication is not mentioned in the manuscript.**

**In item #1 below, report all support for the work reported in this manuscript without time limit. For all other items, the time frame for disclosure is the past 36 months.**

| | | **Name all entities with whom you have this relationship or indicate none (add rows as needed)** | **Specifications/Comments (e.g., if payments were made to you or to your institution)** |
|---|---|---|---|
| | | **Time frame: Since the initial planning of the work** | |
| 1 | All support for the present manuscript (e.g., funding, provision of study materials, medical writing, article processing charges, etc.) **No time limit for this item.** | _X_None | |
| | | | |
| | | | |
| | | | |
| | | | |
| | | | |
| | | | |
| | | | |
| | | **Time frame: past 36 months** | |
| 2 | Grants or contracts from any entity (if not indicated in item #1 above). | _X_None | |
| | | | |
| | | | |
| 3 | Royalties or licenses | __X_None | |
| | | | |
| | | | |
| 4 | Consulting fees | _X_None | |
| | | | |

| | | | |
|---|---|---|---|
| 5 | Payment or honoraria for lectures, presentations, speakers bureaus, manuscript writing or educational events | __X_None | |
| 6 | Payment for expert testimony | __X_None | |
| 7 | Support for attending meetings and/or travel | __X_None | |
| 8 | Patents planned, issued or pending | _X_None | |
| 9 | Participation on a Data Safety Monitoring Board or Advisory Board | _X_None | |
| 10 | Leadership or fiduciary role in other board, society, committee or advocacy group, paid or unpaid | __X_None | |
| 11 | Stock or stock options | __X_None | |
| 12 | Receipt of equipment, materials, drugs, medical writing, gifts or other services | _X__None | |
| 13 | Other financial or non-financial interests | __X_None | |

**Please place an “X” next to the following statement to indicate your agreement:**

**_X I certify that I have answered every question and have not altered the wording of any of the questions on this form.**

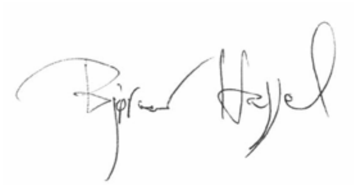

**ICMJE DISCLOSURE FORM**

**Date: February 19th, 2021**
**Your Name: Hege Christensen**
**Manuscript Title:** Clinical application of intrathecal gadobutrol for assessment of cerebrospinal fluid tracer clearance to blood
**Manuscript number (if known):** 147063-INS-CMED-TR-2

**In the interest of transparency, we ask you to disclose all relationships/activities/interests listed below that are related to the content of your manuscript. "Related" means any relation with for-profit or not-for-profit third parties whose interests may be affected by the content of the manuscript. Disclosure represents a commitment to transparency and does not necessarily indicate a bias. If you are in doubt about whether to list a relationship/activity/interest, it is preferable that you do so.**

**The following questions apply to the author's relationships/activities/interests as they relate to the current manuscript only.**

**The author's relationships/activities/interests should be defined broadly. For example, if your manuscript pertains to the epidemiology of hypertension, you should declare all relationships with manufacturers of antihypertensive medication, even if that medication is not mentioned in the manuscript.**

**In item #1 below, report all support for the work reported in this manuscript without time limit. For all other items, the time frame for disclosure is the past 36 months.**

| | | **Name all entities with whom you have this relationship or indicate none (add rows as needed)** | **Specifications/Comments (e.g., if payments were made to you or to your institution)** |
|---|---|---|---|
| **Time frame: Since the initial planning of the work** | | | |
| 1 | All support for the present manuscript (e.g., funding, provision of study materials, medical writing, article processing charges, etc.) **No time limit for this item.** | __X__None | |
| | | | |
| | | | |
| | | | |
| | | | |
| | | | |
| | | | |
| | | | |
| **Time frame: past 36 months** | | | |
| 2 | Grants or contracts from any entity (if not indicated in item #1 above). | __X__None | |
| | | | |
| | | | |
| 3 | Royalties or licenses | __X__None | |
| | | | |
| | | | |
| 4 | Consulting fees | __X__None | |
| | | | |

| | | | |
|---|---|---|---|
| 5 | Payment or honoraria for lectures, presentations, speakers bureaus, manuscript writing or educational events | __X__None | |
| | | | |
| | | | |
| 6 | Payment for expert testimony | __X__None | |
| | | | |
| | | | |
| 7 | Support for attending meetings and/or travel | __X__None | |
| | | | |
| | | | |
| 8 | Patents planned, issued or pending | __X__None | |
| | | | |
| | | | |
| 9 | Participation on a Data Safety Monitoring Board or Advisory Board | __X__None | |
| | | | |
| | | | |
| 10 | Leadership or fiduciary role in other board, society, committee or advocacy group, paid or unpaid | __X__None | |
| | | | |
| | | | |
| 11 | Stock or stock options | __X__None | |
| | | | |
| | | | |
| 12 | Receipt of equipment, materials, drugs, medical writing, gifts or other services | __X__None | |
| | | | |
| | | | |
| 13 | Other financial or non-financial interests | __X__None | |
| | | | |
| | | | |

**Please place an "X" next to the following statement to indicate your agreement:**

**__X_ I certify that I have answered every question and have not altered the wording of any of the questions on this form.**

## ICMJE DISCLOSURE FORM

**Date: February 19th, 2021**
**Your Name: Markus Herberg Hovd**
**Manuscript Title:** Clinical application of intrathecal gadobutrol for assessment of cerebrospinal fluid tracer clearance to blood
**Manuscript number (if known):** 147063-INS-CMED-TR-2

**In the interest of transparency, we ask you to disclose all relationships/activities/interests listed below that are related to the content of your manuscript. "Related" means any relation with for-profit or not-for-profit third parties whose interests may be affected by the content of the manuscript. Disclosure represents a commitment to transparency and does not necessarily indicate a bias. If you are in doubt about whether to list a relationship/activity/interest, it is preferable that you do so.**

**The following questions apply to the author's relationships/activities/interests as they relate to the current manuscript only.**

**The author's relationships/activities/interests should be defined broadly. For example, if your manuscript pertains to the epidemiology of hypertension, you should declare all relationships with manufacturers of antihypertensive medication, even if that medication is not mentioned in the manuscript.**

**In item #1 below, report all support for the work reported in this manuscript without time limit. For all other items, the time frame for disclosure is the past 36 months.**

| | | **Name all entities with whom you have this relationship or indicate none (add rows as needed)** | **Specifications/Comments (e.g., if payments were made to you or to your institution)** |
|---|---|---|---|
| | | **Time frame: Since the initial planning of the work** | |
| 1 | All support for the present manuscript (e.g., funding, provision of study materials, medical writing, article processing charges, etc.) **No time limit for this item.** | __X__None | |
| | | | |
| | | | |
| | | | |
| | | | |
| | | | |
| | | | |
| | | | |
| | | **Time frame: past 36 months** | |
| 2 | Grants or contracts from any entity (if not indicated in item #1 above). | __X__None | |
| | | | |
| | | | |
| 3 | Royalties or licenses | __X__None | |
| | | | |
| | | | |
| 4 | Consulting fees | __X__None | |
| | | | |

| | | | |
|---|---|---|---|
| 5 | Payment or honoraria for lectures, presentations, speakers bureaus, manuscript writing or educational events | __X__None | |
| | | | |
| | | | |
| 6 | Payment for expert testimony | __X__None | |
| | | | |
| | | | |
| 7 | Support for attending meetings and/or travel | __X__None | |
| | | | |
| | | | |
| 8 | Patents planned, issued or pending | __X__None | |
| | | | |
| | | | |
| 9 | Participation on a Data Safety Monitoring Board or Advisory Board | __X__None | |
| | | | |
| | | | |
| 10 | Leadership or fiduciary role in other board, society, committee or advocacy group, paid or unpaid | __X__None | |
| | | | |
| | | | |
| 11 | Stock or stock options | __X__None | |
| | | | |
| | | | |
| 12 | Receipt of equipment, materials, drugs, medical writing, gifts or other services | __X__None | |
| | | | |
| | | | |
| 13 | Other financial or non-financial interests | __X__None | |
| | | | |
| | | | |

**Please place an "X" next to the following statement to indicate your agreement:**

**__X_ I certify that I have answered every question and have not altered the wording of any of the questions on this form.**

## ICMJE DISCLOSURE FORM

**Date: February 19th 2021**
**Your Name: Geir Ringstad**
**Manuscript Title:** Clinical application of intrathecal gadobutrol for assessment of cerebrospinal fluid tracer clearance to blood
**Manuscript number (if known):** 147063-INS-CMED-TR-2

**In the interest of transparency, we ask you to disclose all relationships/activities/interests listed below that are related to the content of your manuscript. "Related" means any relation with for-profit or not-for-profit third parties whose interests may be affected by the content of the manuscript. Disclosure represents a commitment to transparency and does not necessarily indicate a bias. If you are in doubt about whether to list a relationship/activity/interest, it is preferable that you do so.**

**The following questions apply to the author's relationships/activities/interests as they relate to the current manuscript only.**

**The author's relationships/activities/interests should be defined broadly. For example, if your manuscript pertains to the epidemiology of hypertension, you should declare all relationships with manufacturers of antihypertensive medication, even if that medication is not mentioned in the manuscript.**

**In item #1 below, report all support for the work reported in this manuscript without time limit. For all other items, the time frame for disclosure is the past 36 months.**

| | | **Name all entities with whom you have this relationship or indicate none (add rows as needed)** | **Specifications/Comments (e.g., if payments were made to you or to your institution)** |
|---|---|---|---|
| | | **Time frame: Since the initial planning of the work** | |
| 1 | All support for the present manuscript (e.g., funding, provision of study materials, medical writing, article processing charges, etc.) **No time limit for this item.** | Department of Neurosurgery, Oslo University Hospital-Rikshospitalet, Oslo, Norway | |
| | | University of Oslo | Blood Gd analysis |
| | | Norwegian Institute for Air Research, Kjeller, Norway | Blood Gd analysis |
| | | **Time frame: past 36 months** | |
| 2 | Grants or contracts from any entity (if not indicated in item #1 above). | None | |
| | | | |
| | | | |
| 3 | Royalties or licenses | None | |
| | | | |
| | | | |
| 4 | Consulting fees | None | |
| | | | |

| | | | |
|---|---|---|---|
| 5 | Payment or honoraria for lectures, presentations, speakers bureaus, manuscript writing or educational events | Bayer AG, Berlin | Received a Speaker Fee |
| | | | |
| | | | |
| 6 | Payment for expert testimony | None | |
| | | | |
| | | | |
| 7 | Support for attending meetings and/or travel | None | |
| | | | |
| | | | |
| 8 | Patents planned, issued or pending | US Patent Pending | US Patent App. 16/577,227, 2020 |
| | | | |
| | | | |
| 9 | Participation on a Data Safety Monitoring Board or Advisory Board | None | |
| | | | |
| | | | |
| 10 | Leadership or fiduciary role in other board, society, committee or advocacy group, paid or unpaid | X | Chairman of BrainWideSolutions AS |
| | | | |
| | | | |
| 11 | Stock or stock options | X | Shareholder in BrainWideSolutions AS |
| | | | |
| | | | |
| 12 | Receipt of equipment, materials, drugs, medical writing, gifts or other services | None | |
| | | | |
| | | | |
| 13 | Other financial or non-financial interests | None | |
| | | | |
| | | | |

**Please place an "X" next to the following statement to indicate your agreement:**

**X I certify that I have answered every question and have not altered the wording of any of the questions on this form.**
